# Diaspora Policies, Consular Services and Social Protection for Czech Citizens Abroad

**DOI:** 10.1007/978-3-030-51245-3_7

**Published:** 2020-10-31

**Authors:** Eva Janská, Kristýna Janurová

**Affiliations:** 1 FRS-FNRS & Centre for Ethnic and Migration Studies (CEDEM), University of Liege, Liege, Belgium; 2 Centre for Ethnic and Migration Studies (CEDEM), University of Liege, Liege, Belgium; Charles University, Prague, Czechia

## Abstract

The Czech diaspora counts approximately 2.5 million people with Czech origins, including some 912,000 people born in Czechia. Although the diaspora has not been of crucial concern to Czech authorities and political parties in the past decades, a greater interest can be seen in recent years. This chapter is a first attempt at presenting a consolidated overview of the institutional and policy structure towards the Czech diaspora in various areas, showing that Czechia’s engagement with its nationals abroad centers on cultural and educational policies and involves an extension of voting rights, while consular services are conventional and social protection is considered to be primarily the task of the migrants’ host countries. Some exceptions are noted in the areas of health care and pensions. This is being explained by the fact that emigration has not been perceived as a threat to the Czech economy in the past few decades, and the observation that many of the policies introduced came out from bottom-up efforts.

## Introduction

Research on the Czech diaspora is still rather limited. The existing studies are mostly qualitative and focus on questions of ethnic identification, migration and return migration potential after Czechia’s accession to the European Union (EU) (e.g. Vavrečková 2006), migration motives (e.g. Pařízková 2011b), or integration and transnationalism (e.g. Pařízková 2011a; Janurová 2018). Brouček et al. (2017) made the first attempt for a broader characterisation of the contemporary Czech diaspora in a few selected countries, focusing on its needs and relationship with the Czech state. However, no study so far analysed comprehensively the home country policies related to social protection, consular protection, or political participation of the diaspora. This chapter aims to fill this gap. By providing an overview of key institutions and policies, it shows that targeting the diaspora has not been of crucial concern to the Czech authorities and political parties in the past decades, especially in the field of social protection. This attitude may have been influenced by several factors. This includes the steadily low emigration rate (which did not lead to serious fears of brain drain or a weakening of the labor force); the possible mutual reluctance about (re)forming relations with the diaspora after 40 years of communism during which emigrants were persecuted and denigrated; and a general *laissez-faire* attitude to migration in the first decade after the fall of the communist regime in 1989, when the political scene was going through an overall transformation (Baršová and Barša 2005; Drbohlav et al. 2010; Nešpor 2002).

In this chapter, we will show that Czechia’s engagement with its diaspora centers on cultural and educational policies and involves some extension of voting rights, while consular services are conventional and social protection is primarily considered the task of migrants’ host countries. After providing basic information about the structure and geographical distribution of the Czech diaspora, the chapter outlines the general institutional and policy structure that frames the country’s engagement with its nationals abroad in various areas, especially consular protection, education, national elections and culture. Second, it discusses the services provided to nationals abroad in the area of social protection, building on the relevant policies.

## Diaspora Characteristics and Home Country Engagement

### The Czech Diaspora and Its Relations with the Homeland

The available counts of Czech nationals abroad rely on estimates of Czech diplomatic missions abroad, which usually draw on a mixture of sources (host country government statistics, own estimates, research, etc.). In some of them, it is hard to differentiate between people of Czech and Slovak origins due to their historical cohabitation in one state and different methods used to count people of various national or ethnic origins.

According to estimates, the Czech diaspora includes 2.5 million people with Czech origins (including the offspring of people who migrated in the previous centuries), including 912,000 people born in Czechia, which corresponds to 8.5% of the population of Czechia.^1^ The key destination countries are the United States of America (USA, with around 1.3 million inhabitants with Czech and 300,000 with Czechoslovak ancestry in 2018),^2^ Canada (approximately 105,000 people with Czech origins and 40,000 with Czechoslovak origins according to the 2016 census)^3^ and Germany (approximately 190,000 people with a Czech “migration background” in 2018).^4^ In Europe, two other important destinations are the United Kingdom (around 55,000 inhabitants born in Czechia, which mostly overlaps with 49,000 of those who reported Czech nationality, in 2017)^5^ and Austria (14,000 Czech nationals in 2020).^6^ Slovakia, with around 35,000 people speaking Czech as their mother tongue, is another important destination country, although this is mostly due to its long coexistence with Czechia in one state, which enabled a lot of (internal) migration in both directions, including return migration of Slovaks who obtained Czech citizenship.^7^

The reasons for emigration have been changing over the years, being primarily political (before and during the World Wars; during the period of communism from 1948 to 1989) and economic (between the World Wars; in the 1980s period of weakening of the regime and worsening of the economic situation in the home country; and since the 1990s onwards) (Jirásek 1999; Nešpor 2002). Pursuing studies abroad is assumed to be of growing importance as a migration driver since the 1990s and, especially so, since Czechia’s accession to the EU in 2004.

The few studies focused on Czech emigration show that many Czech migrants prefer temporary mobility targeted at the accumulation of financial capital, language skills or international experience, intended to be used as assets for one’s social or economic mobility in Czechia after return (Pařízková 2011a; Vavrečková and Hantak 2008).

In the eyes of Czech authorities, Czech migrants do not face great integration difficulties, this being one potential reason for which they have not introduced or adjusted many policies to suit the day-to-day needs of the diaspora. Yet, state authorities are very supportive of the restoration and promotion of the cultural heritage of Czech(oslovak) diaspora communities, and of the conservation of the knowledge of Czech and Czechia among emigrants and their offspring (perhaps – but not only – with a possible outlook for the benefits of their potential return). Hence, it is worth asking whether a consolidated Czech diaspora policy encompassing a wider spectre of activities actually exists (cf. Brouček 2015; Brouček et al. 2017). The policies discussed in this chapter have to be read with this context in mind.

### Diaspora Infrastructure

In Czechia, no authority focuses exclusively on emigration or diaspora issues, although individual ministries do have departments or offices dedicated to or (also) dealing with diaspora affairs, in addition to other institutions targeting the diaspora (see also Brouček et al. 2017).

The consular departments of the Czech representative authorities abroad, arched over by the Consular Department of the Ministry of Foreign Affairs of the Czech Republic (MFA), provide some of the key official services to Czechs abroad. These include the issue or renewal of passports and birth/death/marriage certificates^8^; help in specific situations of need (illness, accidents, arrest or custody, limitation of personal freedom, loss of travel documents or of financial means, death, natural and human made disasters).^9^ Ambassadors and consuls are also encouraged to develop and maintain ties with the diaspora and contribute to its networking.

Honorary Consular Officers perform some basic consular functions, including help to Czechs in need, assistance and protection in cases of accidents, situations of threat to life, health or property, arrest, retention, sentencing, or death. They also take on some administrative functions, such as facilitating the issue of or changes in travel documents and accepting Czechs’ valuables into custody. Like ambassadors and consuls, they promote social life and support the Czech diaspora.^10^

The Office of the Special Envoy for Expatriate Affairs of the Ministry of Foreign Affairs (*Oddělení zvláštního zmocněnce pro krajanské záležitosti Ministerstva zahraničích věcí České republiky*) was established in 1990 as a Centre for Non-Governmental Relations. It deals with diaspora questions, strengthening relations with Czechs abroad to boost international and economic relations with the host countries, and to deepen and/or support their interest in domestic affairs and in the Czech culture. It also aims to support a positive perception of the diaspora in the homeland. The Office promotes cooperation with diaspora organizations (“societies”), including the local branches of the so-called Czech School Without Border (*Česká škola bez hranic*), through the provision of financial donations for their projects, for the maintenance and repairs of their estate, cultural facilities and small monuments. The Office also supports an educational programme aimed at teaching Czech especially, but not only, to members of the diaspora. It also provides for the issuing of certificates of belonging to the Czech community living abroad, which can be used as a supporting document when applying for permanent residence in Czechia. Additionally, it runs an information service for Czech diaspora organizations around the world and aims at pooling the activities of other national bodies working on diaspora issues – most recently, through the introduction of the Inter-Ministerial Commission for Czechs Living Abroad.^11^

The Standing Senate Commission on Compatriots Living Abroad (*Stálá komise Senátu pro krajany žijící v zahraničí*), established in 1996, acts as an advisory body to the Senate of the Czech Republic, focusing exclusively on issues related to the diaspora, such as postal voting, correspondence issuing of passports or the strengthening of the mutual dialogue between Czechs abroad and those in Czechia.^12^ The Commission meets regularly and maintains contact with diaspora organizations and representatives by e-mail, video conferences and face-to-face meetings in their host countries or in Czechia. It initiates parliamentary discussions about amendments of laws that affect the diaspora, while also participating in diaspora-related expert conferences and roundtables. It collaborates with the Ethnological Institute of the Czech Academy of Sciences, which conducts academic research on the diaspora, and the International Coordination Committee of Czechs Living Abroad (see below). The Commission’s achievements include initiating the legislative change which allowed dual citizenship in 2014; enabling nationals abroad to collect newly issued passports at honorary consulates in 2015; and initiating a law amendment which made some conditions for the Czech School Without Borders comparable to primary schools in Czechia (by removing, for instance, the need for pupils of these schools to do comparative exams in Czech schools). The Commission has established its own Consultative Board as an advisory body consisting of representatives of the state administration and diaspora members.

In 2017, an Inter-ministerial Commission for Czechs Living Abroad (*Meziresortní komise pro Čechy žijící v zahraničí*) was established as an advisory body to the Office of the Special Envoy for Expatriate Affairs of the MFA. It aims to improve information-sharing and cooperation between public authorities (especially ministries) and other public institutions on issues pertaining to Czechs abroad, and gradually create a unified information “database” (in the form of a website^13^) enabling Czechs abroad to deal more easily with administrative, financial and other issues in Czechia, also with respect to their potential return. The Commission shall also submit recommendations and suggestions on measures targeted at improving the relations of the Czech authorities with nationals abroad.

The Czech Centres (*Česká centra*) are a contributory organisation of the MFA, promoting Czechia abroad.^14^ The network is active in public diplomacy abroad, interconnects cultural presentations and supports external economic relations and tourism. The Centres also serve as a meeting place for Czechs abroad, providing an opportunity to keep in touch with Czechia through cultural projects. In 1949, the first “Cultural and Information Centres” were established in Warsaw and Sofia. Gradually, a worldwide network developed, known as “Czech Centres” since 1994.

The International Coordination Committee of Czechs Living Abroad (*Mezinárodní koordinační výbor zahraničních Čechů*) is a civic association founded in 2003. Although it is not a public institution, it serves as a platform for meetings and dialogue between Czechs living in different parts of the world, as well as with people residing in Czechia. The Committee organises gatherings, conferences, exhibitions, publications, the regular “Important Czech Woman in the World” award and other events. The bi-annual International Compatriot Conference, taking place in Prague, is a large event frequented by a number of diaspora representatives, the public administration and academics.^15^

In Czech, the historical term “compatriot” (*krajan*^16^), used for both Czech citizens living abroad and all other people with Czech origins (or who identify themselves as such), has become the most widely used label for the diaspora in (expert) discourse.^17^ For instance, the label is used as such in the Czech title of the Office of the Special Envoy for Expatriate Affairs, the Standing Senate Commission, the International Compatriot Conference, numerous Czech(oslovak) diaspora organizations, etc. Although it is still used even by members of the diaspora themselves, there has been some debate in the past years about its old-fashioned nature and its prevailing connotation of political exiles or emigrés from Nazi or Communist Czechoslovakia. The Standing Senate Commission has thus started a discussion about its potential change of title to one which would denote both people with historical Czech origins, as well as the contemporary Czech diaspora.^18^ The Office of the Special Envoy also prefers using the expression “Czechs abroad” (*Češi v zahraničí*) as an umbrella term for all people of Czech origins living abroad. Some experts and diaspora members use a variant of this term, “foreign Czechs” (*zahraniční Češi*). The widely used international term “diaspora” is however also being more and more commonly used in the Czech context (for instance, in the titles of some of recent conferences and seminars organized by the Standing Senate Commission). This shows that at least for relevant stakeholders in Czechia, “diaspora issues” represent a live topic whose content has evolved in time, along with other political and cultural changes in the societies concerned.

### Key Engagement Policies

After the turn of the regime in the 1990s, the Czechoslovak and later^19^ the Czech Republic took some measures to support the renewal of ties with Czechs (and Slovaks) who had emigrated during the communist period. Besides the formation of cultural and (semi-)diplomatic links via the Ministry of Foreign Affairs, in 1993, the government also enabled emigrants to re-gain the Czech(oslovak) citizenship, which they had frequently been forced by the previous regime to give up (Černý and Valášek 1996).^20^ In 2014, the Czech Republic officially introduced the possibility of dual citizenship, thus reacting, among other things, to the rising number of Czechs living abroad and/or in multi-national families.^21^ This legal change was initiated by the Standing Senate Commission. Apart from that, official relations with Czechs abroad have been limited to standard consular protection, which applies in the same way to citizens permanently residing abroad and temporary stayers. No overall strategy addressing the needs of the diaspora has been introduced, reflecting the fact that there has been no general need to lobby for or protect citizens’ economic, social, cultural or political rights abroad.

The most visible diaspora engagement policies have been in the fields of education, culture, and voting abroad. The possibility to vote in general elections at representative authorities for Czechs residing or staying abroad was used for the first time in the 2012 parliamentary elections.^22^ Apart from elections for the Chamber of Deputies (the Lower House), Czechs abroad can also vote in presidential elections since 2013, when general presidential elections were introduced in Czechia. It is not possible to vote in regional, Senate or European Parliament elections when abroad, but in the latter two types of elections, in-country voting is still possible using voter cards (*voličský průkaz*). General elections can only be held by “professional representative authorities” (embassies, consulates, general consulates and consular offices,^23^ i.e. not honorary consulates or permanent missions). To vote abroad, Czech citizens either have to be registered in the “special list of a representative authority” (registry of voters living abroad) held by the representative authority of the country of residence^24^; or have a voter card issued by the Czech municipality where they have the permanent residence (if applicable)^25^ or by the representative authority where they have until then been registered in a special list.^26^ In the latter case, the voter card can be used to vote in Czechia or at any other representative authority where elections are being held. Voters are obliged to vote in person, but geographical distance is a reason why only a minority of the potential Czech voters residing abroad take the opportunity to do so.^27^ Although their number has been rising, the introduction of postal or electronic voting could contribute to its further growth.^28^ Having been refused three times by the Chamber of Deputies in the past years, correspondence voting is currently part of a bill introduced by the Ministry of the Interior that is expected to pass in 2020.^29^ It is anticipated that the 2021 elections for the Czech Chamber of Deputies will already allow for correspondence voting.

Education is another major field of Czechia’s official engagement with the diaspora. Through its educational programme, organized in cooperation with the Ministry of Education, Youth and Sports of the Czech Republic, the Office of the Special Envoy supports a number of educational activities targeted at the diaspora.^30^ They include the regular summer Czech course for (descendants of) Czechs living abroad, organized by the Charles University^31^; one- and two-semester Czech language courses at public universities in the Czech Republic; Czech language teaching course for teachers from the local communities of Czechs living abroad; sending teachers to Czech communities abroad or sending lecturers of Czech Language and Literature to universities and other institutions in different countries. Teaching of the Czech language, geography and history abroad is currently widely done also by the Czech School Without Borders, which is a growing network of local NGOs established and run – to a large extent on a voluntary basis – by the diaspora in order to maintain and develop their children’s knowledge of the home country culture. Some of the teachers are members of the community, some get involved through the educational programme.^32^

In the cultural field, Czechia engages with its diaspora in numerous ways. The Czech Centres serve as representative spaces both for Czechia-based artists to present their work abroad, and for artists from the diaspora who wish to publicize their activities in Czechia or elsewhere. They also function as a meeting hub. The MFA also engages with the diaspora through support provided to diaspora societies for their cultural projects (maintenance of libraries or memorials, traditional dancing and singing clubs, etc.), and through the (co-)organization of events where diaspora members meet and present their activities to the Czech public.^33^ This includes, for instance, the annual International Compatriotic Festival (organized by the civic association *Občanské sdružení Sedm paprsků*), which is a presentation and competition of diaspora traditional dancing groups^34^; or the annual *Gratias Agit* award ceremony, where individuals or organizations representing the diaspora are awarded by the Minister of Foreign Affairs for spreading Czechia’s good name in the world.^35^ The MFA also financially and/or personally partakes in activities organized by the International Coordination Committee of Czechs Living Abroad. Last but not least, Czechia also engages with the diaspora through its Radio Prague international broadcasting service. This is a channel of the Czech Radio public broadcaster, which transmits news from Czechia in six languages, and is accessible around the world, both on the radio and online.

Repatriation and resettlement also need to be discussed as areas of engagement with the diaspora. When there is need for repatriation (including the repatriation of human remains), the Czech representative authorities provide only basic help, such as informing the respective persons or their relatives, issuing necessary documents (e.g. a cover sheet for the repatriation of human remains from countries which are not subject to relevant international treaties), and facilitating contact with institutions in the home or host country (courts, lawyers, public authorities, hospitals, insurance companies, etc.).^36^ The policy does not distinguish between Czechs permanently and temporarily staying abroad.

Voluntary collective resettlement of nationals or evacuation (or members of the diaspora who may not necessarily have Czech citizenship) takes place in exceptional circumstances, based on government resolutions taken usually in reaction to specific political circumstances (wars, conflicts) or natural disasters. Nowadays, the Ministry of Interior and the Ministry of Foreign Affairs take care of them. The first two resettlement programmes took place in 1946–1947 (resettlement of nationals to vacated areas of the Czechoslovak borderlands) and in 1991–1993 (evacuation of around 2000 nationals from areas in Ukraine and Belarus affected by the Chernobyl nuclear disaster). The third (1995–2001) and the fourth programme (2007) led to the resettlement of the diaspora from Kazakhstan, Russia, Uzbekistan, Kyrgyzstan, and Moldova. Recently, evacuations from Ukraine in 2015, from Libya in 2011 and from Lebanon in 2006 took place, due to political unrest in these countries and extreme life-threatening situations.^37^ The most recent repatriations took place in the spring of 2020 after the outbreak of the covid-19 global pandemic.^38^

Apart from the described areas, there are no other specific policies of engagement, beyond regular procedures formulated by international or European law. Likewise, Czechia has no specific policies targeting the diaspora in the fields of remittances, housing or business. Apart from the reasons already mentioned (low emigration rate and its negligible effect on the Czech economy), the content of Czechia’s engagement with the diaspora might have also been structured by the demand expressed by representatives of the diaspora themselves.

## Diaspora Policies and Social Protection in Czechia

Czechia does not have a social protection strategy for its citizens abroad. Generally, social protection is understood as part of the role of the state of residence. However, entitlements to social allowances deriving from social or sickness insurance may take into account insurance periods spent in Czechia, based on bilateral agreements with third countries (Brouček et al. 2017, p. 77–80; Kropáčová 2014). Nationals residing abroad are thus entitled to social allowances primarily via the institute of aggregation of insurance periods, which is mostly used in the case of pensions, the calculation and payment of which is more complex than in the case of other benefits responding to relatively short-term life situations (ibid.). Nevertheless, if Czech migrants retain their permanent residence in Czechia, they might, theoretically, also keep their entitlement to (some) other social allowances – in such case, they are not considered as “diaspora”.

Although there is no legal obligation to de-register from permanent residence in Czechia when living abroad, it is assumed that Czechs who decide to settle in another country permanently do so for practical reasons, such as easier access to employment, to opening a bank account, or to the social security in the host country (if they have fulfilled the conditions to register their permanent residence there). On the contrary, some Czech migrants may decide to keep their permanent residence in Czechia if they perceive it as easier or more expedient to use the Czech social protection system, rather than arranging to draw support from the host country. Permanent residence thus plays a greater role than nationality in accessing social protection in cases when the country of origin offers better protection than the country of destination. In the case of Czechia, this claim holds the more so because the Czech social protection law does not target Czech nationals abroad per se, and thus only permanent residence ensures the provision of social benefits. That said, although the legal framework for consular services does not state that consulates should assist Czech nationals abroad in cases when they seek to apply for social benefits in Czechia, this might theoretically occur in specific circumstances in response to citizens considered “in need” and applies to all the areas of social protection mentioned below.^39^

### Unemployment

The Czech state has policies in place targeting the diaspora in situations of serious need (see below), as well as educational policies (as described above), that may indirectly apply to nationals abroad in the situation of unemployment. However, no Czech authority provides assistance specifically in the area of (un)employment to Czech citizens abroad beyond what is provided by the EU framework and bilateral agreements with third countries, some of which account for the migrant’s insurance period in Czechia (e.g. the agreement with Turkey^40^).

### Health Care

Nationals who reside abroad permanently are expected to register with a health insurance institution in the country of residence.^41^ However, as stated in European regulations,^42^ permanent migrants can also file an official request to the regional office of the Czech Social Security Administration (CSSA, *Česká správa sociálního zabezpečení*) for an exception from the legal rules, if they for some reason wish to keep their health insurance in Czechia.^43^ If the concerned authorities in Czechia (CSSA and the Health Insurance Bureau (*Kancelář zdravotního pojištění*) and in the host country do not object to the request, the migrant can stay insured in Czechia, provided he/she pays the insurance him-/herself. This exception probably reflects the fact that especially migrants with long-term health problems may prefer to keep undergoing treatment in healthcare facilities where they have already been doing so.

As regards specific policies of healthcare provision to members of the diaspora, the Ministry of Health of the Czech Republic ensures that necessary healthcare, corresponding to the needs of the particular patient, is provided to participants and teachers of internships and teaching or language courses in Czechia, including participants of courses for members of the diaspora.^44^

### Pensions

The pension insurance system covers for old-age, disability, widows’ and widowers’ and orphans’ pensions. These pensions are administered by the Czech Social Security Administration. Nationals abroad can, in line with the relevant international, European and Czech laws, take up their old-age pension benefits based on any legal work contract in any country in which they participated in pension insurance for at least one year, provided they have reached retirement age and have been insured for the needed minimum period stated by the laws of that state.^45^ Other types of pension benefits are also paid out based on the relevant coordination regulations valid for all EU Member States (Kropáčová 2014).

The CSSA and its superior institution, the Ministry of Labour and Social Affairs (*Ministerstvo práce a sociálních věcí České republiky*), are the only Czech authorities active in the field of pensions for nationals abroad. As such, the CSSA provides information and the necessary documents to nationals abroad who seek to claim Czech pensions, and together with the Ministry, they publish basic instructions online and in print in the form of information sheets and guides such as, for instance, the Guide for migrating pensions (*Příručka pro migrující osoby*).^46^

The payment of pension benefits to nationals residing in third countries is regulated by bilateral agreements on social security which are divided into three types.^47^ *Proportional agreements* (e.g. with Austria, Australia, France, Canada, Poland, the USA) are based on the principle of equity, single insurance, aggregation of insurance periods and paying out of the pensions to the second state of agreement. According to these agreements, pensions are paid out by both or all states concerned. *Territorial agreements* are based on the principle of permanent residence of the beneficiary. For example, old-age pensions are paid out by the state of permanent residence even for the period the beneficiary was insured in the other state. This is a historical principle of social security coordination, used in Czechoslovakia’s bilateral agreement with the former Union of Socialist Soviet Republics (USSR). Czechia’s former agreements with the Russian Federation and other follower states of the former USSR were based on this principle, but were terminated in 2008 and 2009, respectively, and replaced with other agreements. *Combined agreements* are proportional with a territorial element, which concerns the evaluation of insurance periods gained before a certain date. For example, in the case of the agreement with Slovakia, this is the date of the dissolution of the Czechoslovak Federal Republic (1 January 1993), whereas for the agreements with Ukraine and Russia, this is the date of the entry into force of the agreement (ibid.). In the case of third countries with which Czechia has not signed bilateral agreements, insurance periods spent in the other country are not taken into account when evaluating the entitlement to pension benefits (Kropáčová 2014).^48^ New agreements are continuously being negotiated and signed (ibid.).

As regards the payment of pensions, “all payments of pension benefits are made through the Czech National Bank in Prague (…) and its contractual partners – its correspondent banks”.^49^ The payment can be carried out in five different ways: by cheque sent to the address of the pension beneficiary abroad (other than in Slovakia^50^ and Canada^51^); directly to the beneficiary’s account at a financial institution abroad; to the beneficiary’s account (or his/her spouse’s account) in the Czech Republic; in the form of a money order to an address in Poland^52^; or to an account in Euro in Slovakia. The conditions for entitlement to payment of a pension benefit are verified by the Certificate of Living, which is a formal document declaring the beneficiary’s living that has to be filled in, signed and the signature officially verified.^53^ The certificate is either sent once per year by the CSSA to the beneficiary’s address abroad to be filled in (in the case of payment by cheque or to an account in Euro in Slovakia) or sent to the CSSA by the beneficiary him-/herself in intervals of their choice, which correspond to the intervals of payment of the pension benefit (in the case of payment to accounts abroad or in Czechia) (ibid.). The Certificate of Living does not have to be submitted by beneficiaries receiving pension to addresses in Poland.

The described variability in the range of bilateral agreements reflects the complexity of the system of pension insurance, which needs to take into account not only the places of former and current residence of the beneficiary, but also all his/her individual employment contracts signed in one or more countries.

### Family-Related Benefits

Family-related benefits available in Czechia include benefits financed from sickness insurance, on the one hand, and benefits financed from the system of “State Social Support”, on the other. Benefits financed from sickness insurance that might be of concern to nationals abroad include the maternity benefit (taken up for 22–37 weeks, beginning just prior or after child birth) and fathers’ post-natal care (effective as of 1 January 2018, it can be taken up for up to a week within 6 weeks of the date of child birth or of the date foster care begins).^54^ Other sickness benefits are tied to employment in Czechia. From the benefits provided under State Social Support, the only one that might be of relevance to nationals abroad, as long as they keep their permanent residence in Czechia, is the parental allowance, because it is drawn on a long-term basis and is not tied strictly to family’s earnings or living costs. This allowance is provided to a parent who personally and duly cares for the youngest child in the family up to 4 years of the child’s age.

Czechia has no special policies regulating the payment of these benefits to nationals abroad. Like in the case of unemployment benefits, for instance, in third countries tied by bilateral agreements with Czechia, the provision of family benefits is primarily seen as the responsibility of the receiving state, in some cases (and if relevant) with reference to the insurance period spent in Czechia. In cases of double eligibility, allowances are provided based on the regulations of the country of residence/later insurance (*Ministerstvo práce a sociálních věcí České republiky*, 2019). Like in the case of pensions, the only authorities that assist nationals abroad in the area of family-related benefits are the CSSA and the Ministry of Labour and Social Affairs of the Czech Republic. The CSSA provides information and the necessary documents to nationals abroad (or those who are about to move abroad), who are eligible for family-related benefits based on their (previous) sickness insurance in Czechia, and together with the Ministry, they publish basic instructions online and in print in the form of information sheets and guides, such as the Guide for migrating pensions. These forms of assistance however do not go beyond the extent of assistance provided to people residing in Czechia.

### Economic Hardship

The System of Assistance in Material Need is regulated by the Act no. 111/2006 Coll., on Assistance in Material Need, with amendments (*Zákon č. 111/2006 Sb., o pomoci v hmotné nouzi, ve znění pozdějších předpisů*). According to this Act, three types of benefits can be paid out to persons in specific situations of material need: the Allowance for Living, the Supplement for Housing and the Extraordinary Immediate Assistance. Czechia has no special policies regulating the payment of these benefits or the provision of other forms of assistance (including to homeless persons) to nationals abroad, beyond policies stated by relevant international coordination regulations.^55^

However, in the case of serious need, representative authorities can provide adequate financial or material support to Czechs residing abroad, if this is indispensable, if it is not possible to obtain such aid by other means, and if the recipient commits to cover the costs of consular protection.^56^ This commitment is not requested in cases of exceptional circumstances. Situations when financial or material aid can be provided are especially such when a citizen’s life is being threatened.

## Conclusions

This chapter shows that the engagement of Czech authorities with the Czech diaspora has been more pronounced in the areas of education and culture, while in the area of social protection, it does not extend beyond the application of basic coordination measures defined by the EU law. This observation reflects the fact that emigration has not been of a threat to the Czech economy in the past few decades, and perhaps that the demand for more intervening policies has not been (perceived as) significant.

Czechia’s engagement with the diaspora in the field of consular protection follows general principles of consular protection applied internationally. Ambassadors and consuls are encouraged to contribute to the networking of the diaspora, but do not provide explicit support to Czech citizens to access benefits in the home or host country.

Teaching of the Czech language, history and geography to diaspora members is one of the key activities supported by the Czech authorities, by way of financial donations to diaspora organisations or support of teachers’ individual postings abroad. Like in the case of education, Czechia’s intensive contact with the diaspora in the cultural field is diverse and long-term, including the Czech Centres; the financial and institutional support to cultural projects and events organized by, for and about the diaspora; and the support to the Czech international broadcasting service. As for political participation, rather recently (2002 and 2013 respectively), Czechs abroad have been made able to take part in parliamentary (Chamber of Deputies) and presidential elections in person at representative authorities.

As regards the five areas of social protection analysed here (unemployment, health care, pensions, family-related benefits and economic hardship), the chapter has shown that Czechia employs no policies beyond the scope of general coordination regulations valid in the EU, and corresponding policies concerning third countries with which Czechia has bilateral agreements. The only specificity includes the variations in how pension benefits are calculated and paid out to nationals living in various parts of the world, depending on historical relations and bilateral agreements (in particular with Slovakia, Poland, Canada and some post-Soviet states). Besides this, Czech nationals abroad have some access to the Czech social protection system only as long as they retain their permanent residence in Czechia. However, in such cases, they are not perceived as “diaspora”.

There have been no major controversies or political debates concerning diaspora’s access to social protection in the recent years, and no specific changes are currently being discussed. The only topics that have recently been negotiated include the introduction of postal voting (this proposal, submitted by the Senate, has been refused repeatedly by the Chamber of Deputies in the past years, but is again part of a bill that is currently in the legislative process) and that of the law amendment allowing dual citizenship, which came into force in 2014.

Overall, this chapter illustrates that the Czech state is not indifferent to Czech nationals abroad, be it in the sense of support to those in need or in a more symbolic sense, meaning that maintenance of contact is considered a valuable cultural resource. This attitude has changed since the 1990s, when official relations with the diaspora were just being (re)formed, while the public perception of (returning) Czech migrants was rather suspicious or straightforwardly critical (Nešpor 2002, 2005). To the contrary, recent years, which brought about the introduction of dual citizenship to Czech law or the establishment of the Inter-ministerial Commission for Czechs Living Abroad, show that diaspora issues are getting more attention from a wider spectre of state authorities than in the past. This chapter shows that a lot of this newly formalized contact between the diaspora and the Czech state came out from bottom-up efforts and activities (e.g. calls for voting from abroad, demands from people thinking about return to have information about the bureaucratic process easily accessible in one place, diaspora cultural and education activities). According to Brouček (2011, p. 51–52), minimal, non-intruding relations to the diaspora are sufficient, since attempts at “organizing the life of Czechs abroad” – e.g. by introducing diaspora-oriented institutions in the home country – do not “pay off”, when migrants themselves do not perceive them as desirable. Migrants who wish to keep relations with one another or with the homeland will always find their ways, depending on their needs. From this perspective, it seems that even though the Czech diaspora policy is not robust and may come off as unconsolidated, it does (aim to) respond to the real needs of those concerned.

## Footnotes

1 See: *Český statistický úřad* (2019). *Obyvatelstvo*. https://www.czso.cz/csu/czso/obyvatelstvo_lide. Accessed 18 March 2020; and *Ministerstvo zahraničních věcí České republiky* (2012). *Češi v zahraničí – číselné údaje.* (Unpublished document.) *Praha: Ministerstvo zahraničních věcí České republiky*; and UN (2019). *International migrant stock 2019*. United Nations Population Division, Department of Economic and Social Affairs. https://www.un.org/en/development/desa/population/migration/data/estimates2/estimates19.asp. Accessed 18 March 2020.

2 United States Census Bureau. *Selected* Population *Profile in the United States. 2018 American Community Survey 1-Year Estimates.* https://data.census.gov/cedsci/table?t=525%20-%20Czech%20%28111-113%29%3A526%20-%20Czechoslovakian%20%28114%29&tid=ACSSPP1Y2018.S0201&hidePreview=false&vintage=2018. Accessed 18 March 2020.

3 Statistics Canada (2018). *Census profile, 2016 census*. https://www12.statcan.gc.ca/census-recensement/2016/dp-pd/prof/details/Page.cfm?Lang=E&Geo1=PR&Code1=01&Geo2=&Code2=& Data=Count&SearchText=Canada&SearchType=Begins&SearchPR=01&B1=All&GeoLevel=PR&GeoCode=01. Accessed 29 September 2018.

4 *Destatis. Statistisches Bundesamt* (2019). *Bevölkerung in Privathaushalten 2018 nach Migrationshintergrund.* https://www.destatis.de/DE/Themen/Gesellschaft-Umwelt/Bevoelkerung/Migration-Integration/Tabellen/migrationshintergrund-staatsangehoerigkeit-staaten.html. Accessed 5 March 2020.

5 Office for National Statistics (2018). *Population of the UK by country of birth and nationality*. January to December 2017. https://www.ons.gov.uk/peoplepopulationandcommunity/populationandmigration/internationalmigration/data sets/populationoftheunitedkingdombycountryofbirthandnationality. Accessed 29 September 2018.

6 *Statistik Austria* (2020). *Bevölkerung zu Jahresbeginn 2002–2020 nach detaillierter Staatsangehörigkeit*. https://www.statistik.at/web_de/statistiken/menschen_und_gesellschaft/bevoelkerung/bevoelkerungsstruktur/bevoelkerung_nach_staatsangehoerigkeit_geburtsland/index.html. Accessed 5 March 2020.

7 Výborný, J. (2012). *České menšiny v SR ubývá*. https://www.cesi.sk/akt/12/nsr.htm. Accessed 30 July 2018.

8 Czech citizen ID cards can only be issued by municipal authorities in Czechia. No other documents (driving license, health insurance card, etc.) can be used as identification documents in Czechia.

9 *Zákon č. 150/2017 Sb., o zahraniční službě a o změně některých zákonů (zákon o zahraniční službě), § 17*

10 *Statut českého honorárního konzulárního úředníka*. (2010). https://www.mzv.cz/file/919209/Statut_CJ.doc. Accessed 1 June 2018.

11 See: *Meziresortní komise pro Čechy žijící v zahraničí* (2017). *Ministerstvo zahraničních věcí České republiky*. https://www.mzv.cz/jnp/cz/zahranicni_vztahy/krajane/meziresortni_komise_pro_cechy_zijici_v/index.html. Accessed 1 June 2018. See also: Ministry of Foreign Affairs of the Czech Republic (2015). *Czechs Abroad*. https://www.mzv.cz/jnp/ en/foreign_relations/czechs_living_abroad/index.html. Accessed 1 June 2018.

12 Standing Senate Commission on Compatriots Living Abroad. Senate of the Parliament of the Czech Republic. http://www.senat.cz/organy/index.php?ke_dni=30.4. 2017&O=11&lng=en&par_2=376. Accessed 5 June 2018.

13 *Užitečné informace pro Čechy žijící v zahraničí*. *Ministerstvo zahraničních věcí České republiky*. https://www.mzv.cz/cesi_v_zahranici/cz/index.html. Accessed 1 June 2018.

14 Czech Centres. *About us*. http://www.czechcentres.cz/en/about-us/. Accessed 5 June 2018.

15 *Mezinárodní koordinační výbor zahraničních Čechů* (2018). http://www.zahranicnicesi.com/. Accessed 5 June 2018.

16 The word “*krajan*” literally means “someone from the same land”.

17 *Ministerstvo zahraničních věcí České republiky* (2017). *Z čeho vychází naše práce? Koncepce vztahu MZV k Čechům v zahraničí.* https://www.mzv.cz/jnp/cz/zahranicni_vztahy/krajane/koncepce_vztahu_mzv_ k_cechum_v_zahranici.html. Accessed 13 July 2018.

18 *Zápis č. 4 z 4. schůze Stálé komise Senátu pro krajany žijící v zahraničí ze dne 20.9. 2017*. 2017. Stálá komise Senátu pro krajany žijící v zahraničí. http://www.senat.cz/xqw/xervlet/pssenat/htmlhled? action=doc&value=85963. Accessed 5 June 2018.

19 After the 1993 dissolution of the country

20 *Zákon č. 193/1999 Sb., o státním občanství některých bývalých československých občanů*

21 *Zákon č. 186/2013 Sb., o státním občanství České republiky a o změně některých zákonů (zákon o státním občanství České republiky)*

22 For further information regarding electoral rights, see: Linek, L. (2018). *Report on political participation of mobile EU citizens: Czech Republic.* Country report 2018/16. Global Citizenship Observatory (GLOBALCIT). Robert Schuman Centre for Advanced Studies in collaboration with Edinburgh University Law School. San Domenico di Fiesole: European University Institute. https://cadmus.eui.eu/handle/1814/59568. Accessed 5 March 2020.

23 “*konzulární jednatelství*” in Czech

24 Application to be registered has to be delivered to the representative authority at least 40 days prior to the elections.

25 There is no legal obligation to de-register from permanent residence in Czechia when living abroad. Hence, some citizens, especially those who aim to return after some time, retain their residence in Czechia, in order to maintain e.g. their Czech health insurance and other legal ties to the home country (see section II).

26 Kandalec, P. (2013). *Access to electoral rights: Czech Republic*. EUDO Citizenship Observatory. http://eu do-citizenship.eu/admin/?p=file&appl=countryProfiles&f=136-Chech-FRACIT.pdf. Accessed 5 June 2018.

27 Vilímová, T. (2017). Půl milionu Čechů v zahraničí má smůlu. Volit mohou jen obtížně. *Echo24.cz* (updated 2 October 2017). https://echo24.cz/a/iz6dw/pul-milionu-cechu-v-zahranici-ma-smulu-volit-mohou-jen-obtizne. Accessed 10 March 2019.

28 In the 2002 parliamentary elections, only around 3,700 Czechs voted abroad. This number increased up to 6,700 in 2006 and 10,500 in 2013 and 2017. More than 7,700 Czechs voted in the 2013 presidential elections from abroad and around 17,500 did so for the 2018 presidential election. See: *Český statistický úřad* (2018). *Výsledky voleb a referend*. https://www.volby.cz/. Accessed 18 July 2018.

29 *Návrh zákona o správě voleb*. Čj. OVA 80/20. *Úřad vlády České republiky*. https://apps.odok.cz/veklep-detail?pid=KORNBH2MHKS9. Accessed 10 March 2020.

30 Program *podpory českého kulturního dědictví na léta 2016–2020 (usnesení vlády)/*Programme of the support of the Czech cultural heritage for 2016–2020 (Government decree).

31 *Letní kurz češtiny pro krajany* (2018). http://www.ubytovani-dobruska.cz/studium-letni-kurz-cestiny-pro-krajany. Accessed 18 July 2018.

32 For further information, see also: *Česká škola bez hranic* (2018). http://csbh.cz/. Accessed 18 July 2018; *Ministerstvo zahraničních věcí České republiky* (2017). *Z čeho vychází naše práce? Koncepce vztahu MZV k Čechům v zahraničí.* https://www.mzv.cz/jnp/cz/zahranicni_vztahy/krajane/koncepce_vztahu_mzv_ k_cechum_v_zahranici. html. Accessed 13 July 2018.

33 *Program podpory českého kulturního dědictví na léta 2016–2020 (usnesení vlády)/*Programme of the support of the Czech cultural heritage for 2016–2020 (government decree).

34 *Mezinárodní krajanský festival* (2017). http://krajanskyfestival.eu/index.php. Accessed 18 July 2018.

35 *Ministerstvo zahraničních věcí České republiky* (2018). *Cena Gratias Agit – úvodní informace*. https://www.mzv.cz/jnp/cz/zahranicni_vztahy/verejna_diplomacie/ceny_gratias_agit/index.html. Accessed 18 July 2018.

36 *Zákon č. 150/2017 Sb., o zahraniční službě a o změně některých zákonů (zákon o zahraniční službě), § 17*

37 *Ministerstvo vnitra České republiky* (2018). *Integration of Compatriots Resettled to the Czech Republic*. http://www.mvcr.cz/webpm/clanek/integration-of-compatriots-resettled-to-the-czech-republic.aspx? q=Y2hudW09Mg%3D%3D. Accessed 30 July 2018.

38 *Ministerstvo zahraničních věcí České republiky* (2020). *Dopis zvláštního zmocněnce krajanům v souvislosti s pandemií Covid-19*. https://www.mzv.cz/jnp/cz/zahranicni_vztahy/krajane/aktualni_informace/dopis_zvlastniho_zmocnence_krajanum_v.html. Accessed 7August 2020.

39 *Zákon č. 150/2017 Sb., o zahraniční službě a o změně některých zákonů (zákon o zahraniční službě), § 17*

40 *Ministerstvo práce a sociálních věcí České republiky* (2019). *Dvoustranné smlouvy o sociálním zabezpečení* (updated 27 October 2019). https://www.mpsv.cz/web/cz/dvoustranne-smlouvy-o-socialnim-zabezpeceni. Accessed 12 March 2020.

41 *Kancelář zdravotního pojištění* (2016). *Kde mám být pojištěn?* https://www.kancelarzp.cz/cs/ pojistenci/prava-naroky-eu/zdr-poj-pece-eu/kde-byt-pojisten. Accessed 30 July 2018.

42 Regulation (EC) No. 883/2004 of the European Parliament and of the Council of 29 April 2004.

43 For example, some people prefer to keep their registration with particular specialist doctors in Czechia (dentists, gynecologists, etc.), and choose to make regular home country visits to have their check-ups done there.

44 *Usnesení vlády České republiky č. 348 z 13. května 2015 o pokračování Programu podpory českého kulturního dědictví v zahraničí na léta 2016 až 2020/* Government Resolution on the Continuation of the Programme to Support the Czech Cultural Heritage Abroad for the Years 2016 to 2020

45 Důchody (2018). *Užitečné informace pro Čechy žijící v zahraničí. Ministerstvo zahraničních věcí České republiky.* https://www.mzv.cz/cesi_v_zahranici/cz/zdravotni_a_socialni_pojisteni_duchod_a/ducho dy/index.html. Accessed 30 July 2018.

46 *Ministerstvo práce a sociálních věcí České republiky* (2012). *Příručka pro migrující osoby*. https://www.mpsv.cz/files/clanky/13776/Prirucka_pro_migrujici_osoby.pdf. Accessed 30 September 2018.

47 *Česká správa sociálního zabezpečení*. *Mezinárodní smlouvy*. https://www.cssz.cz/cz/mezinarodni-smlouvy/. Accessed 28 July 2018.

48 This concerns most African, Asian and South and Central American countries.

49 Czech Social Security Administration. *Conditions for paying out of pension benefits to persons with a permanent place of residence outside the Czech Republic*. https://www.cssz.cz/en/pension-insurance/conditions-for-paying-out-of-pension-benefits-to-persons-with-a-permanent-place-of-residence-outside.htm. Accessed 28 July 2018.

50 The reasons why pensions cannot be paid out to Slovakia in the form of a cheque are historical and geopolitical. After the dissolution of Czechoslovakia in 1993, a special form of automatic monthly payments was introduced for pensioners receiving Czech pension but living in Slovakia, based on their quarterly submission of the Certificate of Living (Employee of CSSA, personal email communication, May 2018).

51 The reason why Czech pensions cannot be paid out to Canadian residents in the form of a cheque is that the Royal Bank of Canada, which is the only correspondent bank in Canada responsible for these payments, no longer produces printed cheques for clients who have accounts in other banks, and therefore cannot guarantee the delivery of the payment to the correct recipient (Employee of CSSA, personal email communication, May 2018).

52 There are historical reasons why Poland is the only country where Czech pensions can be paid out to the recipients’ addresses in the form of a money order. As Poland used to be the biggest recipient of Czechoslovak international payments of pensions in the period 1948–1989, the countries have introduced a system of regular monthly payments conducted via their respective postal services. This system was reinforced by a treaty signed in 2003. Since international money orders can be delivered to the addressee only, Polish recipients of Czech pensions do not have to prove their living (Employee of CSSA, personal email communication, May 2018).

53 *Potvrzení o žití – pro vyplácení důchodu/Certificate of living – for payment of pension*. https://www.cssz.cz/ NR/rdonlyres/D86ED0EC-DF95-45E8-9132-077F98776FA3/0/POZA.pdf. Accessed 30 July 2018.

54 Czech Social Security Administration (2017). *Benefits provided under sickness insurance*. https://www.cssz.cz/en/sickness-insurance/benefits-provided-under-sickness-insurance.htm. Accessed 30 September 2018. See also: Ministry of Labour and Social Affairs. *State Social Support*. https://www.mpsv.cz/en/1603. Accessed 30 September 2018.

55 *Česká správa sociálního zabezpečení*. *Koordinace sociálního zabezpečení*. http://www.cssz.cz/cz/ evropska-unie/socialni-zabezpeceni/. Accessed 20 July 2018.

56 *Zákon č. 150/2017 Sb., o zahraniční službě a o změně některých zákonů (zákon o zahraniční službě)*
